# Celebrating queer chemists

**DOI:** 10.1038/s42004-023-01010-4

**Published:** 2023-09-30

## Abstract

Today, *Communications Chemistry* launches a series of Q&A articles conducted with queer chemists. Here, we discuss the motivation for and aim of this series, and present some key take-home messages from our respondents.

Diversity is a driving force behind innovation, discovery, and progress. Equity and inclusion are pillars of a just and well-rounded society. Despite more than half a century of queer rights activism, equality for people with minority genders and sexual orientations is not a reality. The Human Rights Campaign declared a state of emergency for LGBTQ+ people in the United States on June 6th 2023 — for the first time in the organization’s more than 40-year history^1^. Spurred by these recent disconcerting societal trends, we believe it is imperative that the scientific community lifts up and celebrates our LGBTQ+ colleagues, acknowledges the challenges that queer scientists face, and actively works to address them. With this pledge in mind, *Communications Chemistry* is thrilled to introduce our Queer in Chem series — a collection of Q&A articles in which we interview accomplished queer chemists.

**Figure:**
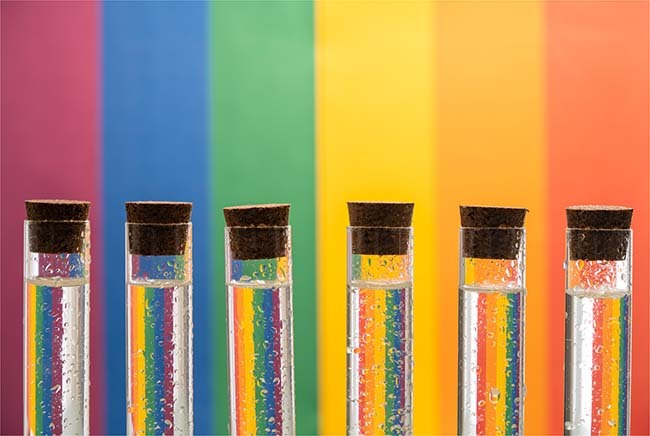
iStock MEDITERRANEAN

These Q&A articles cast a spotlight on queer and trans chemists from various branches of chemistry and with a diverse spectrum of backgrounds, experiences, and perspectives. The series showcases each researcher’s scientific interests as well as their professional experiences. While the questions we asked our respondents revolve around their chemical endeavors, our goal is to depict the people behind the science who often remain invisible. We hope to foster a deeper understanding of the rich tapestry of identities and experiences within the chemistry community. We aim to break down stereotypes and inspire queer chemists to pursue their scientific ambitions. Prof Abhik Ghosh, who we interviewed for the series, shares this goal: “Globally, the overwhelming majority of queer people remain closeted, and I feel I must do my part to give voice to this vast populace that remains voiceless.”

Discrimination and prejudice persist today, creating barriers that can hinder the full potential of LGBTQ+ scientists. “Constantly self-censoring is emotionally exhausting […] This is mental energy that I would rather be spending on chemistry,” says interviewee Prof Polly Arnold. In an effort to improve the reality in which chemical research is done, this Queer in Chem series also aims to provide a forum to discuss what the scientific community as a whole could do to demolish difficulties that LGBTQ+ chemists still face along their career paths. “[Allyship] essentially means leveraging personal privilege to support and uplift others,” says Dr Jovan Dragelj. “Our cisgender and straight allies need to recognize hatemongering for what it is, and to deprive it of the respectability and oxygen that it so desperately craves,” says Prof Nancy Williams. “It’s incredibly heartening when people who aren’t LGBTQ+ are prepared to make their voice heard in defense of their colleagues,” adds Dr Joshua Makepeace.

Our participants also share a host of suggestions for how employers can make a difference for LGBTQ+ scientists. Prof Polly Arnold, Dr Camille Bishop and Prof Nancy Williams comment on the severe challenges that the community faces with respect to (national and) international travel. Dr Jovan Dragelj, Prof Abhik Ghosh and Prof Nancy Williams call out the need for robust mechanisms and effective support for tackling discrimination and harassment in science. Dr Camille Bishop, Dr Jovan Dragelj, Prof Abhik Ghosh and Prof Anna Slater advocate for platforms, safe spaces and networks for marginalized groups to share their experiences. “I’ve benefited enormously from being part of […] a space where I can be more of my ‘full self’. The conversations that can be had – and the explanations that don’t need to happen – are liberating,” shares Prof Anna Slater.

The series also celebrates ways in which queer identity has helped to shape better scientists and better educators. “Sometimes I think my queer identity has actually opened more doors for me. […] I feel that some of that questioning has made me more confident in taking risks in my career,” says Dr Camille Bishop. “The process of having dealt with being gay and coming out to friends and family can make one less afraid to challenge consensus. I’ve long considered myself to have a healthy disrespect for traditional subject boundaries, for example,” says Prof Andrew Goodwin. “Different life experiences can shape the kinds of questions we ask, the approaches we take, and the way we educate and train budding scientists,” adds Prof Nancy Williams.

We extend our heartfelt thanks to the participants who have generously shared their experiences and insights, and encourage you to read each interview in its entirety. By amplifying the voices and experiences of queer chemists, *Communications Chemistry* endeavors to foster a community of understanding, acceptance, and support within the field of chemistry and society at large.
